# Incidence of thyroid cancer in Puerto Rico and the US by racial/ethnic group, 2011–2015

**DOI:** 10.1186/s12885-019-5854-3

**Published:** 2019-06-28

**Authors:** Guillermo Tortolero-Luna, Carlos R. Torres-Cintrón, Mariela Alvarado-Ortiz, Karen J. Ortiz-Ortiz, Diego E. Zavala-Zegarra, Edna Mora-Piñero

**Affiliations:** 1 Puerto Rico Central Cancer Registry, University of Puerto Rico Comprehensive Cancer Center, San Juan, Puerto Rico; 2 Division of Cancer Control and Population Sciences, University of Puerto Rico, Comprehensive Cancer Center, PO Box 70344, San Juan, PR 00936-8344 Puerto Rico; 3 Department of Social Sciences, Graduate School of Public Health, Medical Sciences Campus, University of Puerto Rico, San Juan, Puerto Rico; 4 Department of Surgery, School of Medicine, Medical Sciences Campus, University of Puerto Rico, San Juan, Puerto Rico

**Keywords:** Puerto Rico, Hispanic, Thyroid Cancer, Overdiagnosis, Health disparities

## Abstract

**Background:**

Puerto Rico has the highest incidence rate of thyroid cancer (TC) in the Americas and the third highest rate worldwide. The purpose of this study was to compare the burden of TC between the population of PR and United States (US) non-Hispanic Whites (NHW), non-Hispanic Blacks (NHB), and US Hispanics (USH) during the period 2011–2015.

**Methods:**

TC data for the period 2011–2015 was obtained from the Puerto Rico Central Cancer Registry (PRCCR) and the Surveillance Epidemiology and Ends Results Program (SEER) 18 Registries Research Data. TC was categorized in: papillary carcinoma (PTC), and other TC histologic types. Data was analyzed by sex, age groups, and histologic type. Racial/ethnic differences by sex, age, and histologic types were assessed using the Standardized Rate Ratio (SRR) and its 95% CI.

**Results:**

During the period 2011–2015 there were 5175 and 65,528 cases of TC diagnosed in PR and the US, respectively. The overall age-adjusted incidence rate of PTC was almost two-fold higher in PR than in the US (25.8/100,000 vs. 12.9/100,000). Among PR women, the incidence rate of PTC was 40.0/100,000 compared to 19.4/100,000 in US. PR women had 83% increased risk of being diagnosed with PTC than NHW women, a 2.25-fold increased risk than USH, and 3.45-fold increased risk than NHB women. For men, PR had 34% increased risk of being diagnosed with PTC than NHW men, 2.2-fold increased risk than USH men, and 3.2-fold higher risk than in NHB men.

**Conclusion:**

Further research is needed to understand this disparity in the island. This research should address the extent of overdiagnosis in PR, the role of health insurance status and insurance type, characteristics of the healthcare delivery system as well as the role of patient and environmental factors.

## Background

Based on GLOBOCAN 2018 [1], Puerto Rico (PR) has the highest incidence rate of thyroid cancer (TC) in the Americas and the 4th highest rate worldwide, just below the Republic of Korea, Cyprus and Canada. Whereas, the continental United States (US) ranks 2nd in the Americas and 7th in worldwide. In PR TC is the 3rd leading cancer in women and 13th in men, accounting for 11.5 and 2.5% of all cancers diagnosed in women and men, respectively [2]. The overall average age-adjusted incidence rate of invasive TC in the US for the period 2011–2015 was 14.4/100,000; whereas, in PR it was 27.3/100,000. Similar to other populations, in PR mortality rates from thyroid cancer are very low. The overall age-adjusted mortality of TC in PR for the period 2011–2015 was 0.3/100,000. In addition, during the period 1987 to 2015 mortality rates from thyroid cancer declined 1.2% per year in women and 1.6% per year in men.

A rapid increase in the incidence of TC in the last decades is well documented worldwide [3–7]. This trend has been observed in both, men and women; however, the incidence rates of TC have been consistently higher in women than in men and the increase has been restricted to papillary thyroid cancer (PTC) [5, 8–10]. In the US, TC is the most rapidly increasing cancer in both women and man. TC incidence in the US increased from 10.9/100,000 in 2000 to 21.6/100,000 in 2015 in women and from 3.8/100,000 to 7.5/100,000 in men (Surveillance, Epidemiology, and End Results (SEER) Program SEER*Stat Database: Incidence - SEER 18 Regs). Similarly, in PR TC was the most rapidly increasing cancer during the period 2000 to 2015; TC rates increased from 7.7 to 50.7/100,000 in women and 2.4 to 13.7/100,000 in men (PRCCR, 2018).

The increasing trend in the incidence of invasive TC is partially attributed to overdiagnosis of small lesions due to: increasing use of new diagnostic modalities [11–17]; increasing medical surveillance [11–13, 18]; and increasing access to healthcare services [19–21]. Vaccarella et al., [22] estimated that during the period 2003–2007, in selected industrialized countries, 51–83% of cases diagnosed in women and 3–59% of cases diagnosed in men could be attributed to overdiagnosis. In addition to overdiagnosis, other factors found associated with TC risk include: radiation exposure from computer tomography scans; nutritional, menstrual and reproductive factors; obesity [23]; insulin resistance; physical inactivity [24]; and high socioeconomic status [19, 21, 25].

Racial/ethnic difference in the incidence of TC in the US and other populations are reported [26–31]. In the US, higher incidence rates of TC are observed among Non-Hispanic Whites (NHW); intermediate rates among US Hispanics (USH); and the lowest among Non-Hispanic Blacks (NHB). Puerto Ricans constitute the second largest Hispanic population in the US with more than 4 million living in the continental US and 3.5 million living in the Commonwealth of PR. In PR, 99% of the population self-identify as Hispanics of whom 97% are PR born; women account for 52.5% of the population; and the median age of population is 40 years [32, 33]. Forty-five percent of the population of PR live below the federal poverty line, compared with 16% in US. This is two-times higher the percentage reported in the poorest state in the US, Mississippi (22%). The median annual income in PR is $19,630 (US $51,915) and 60% (US 17%) have a household income of <$25,000 [32, 33]**.**

To our knowledge, no study has compared the incidence of TC between PR residents and US racial/ethnic groups; therefore, the purpose of this analysis is to compare for the first time the incidence of TC in PR with that observed in NHW, NHB, and USH by age and gender using data from the Puerto Rico Central Cancer Registry (PRCCR) and the Surveillance Epidemiology End Results Program (SEER) for the period 2011–2015, the latest period available for both data sources.

## Methods

We obtained TC incidence data for the period 2011–2015 from the PRCCR [2] and the SEER 18 Registries database [34]. The National Cancer Institute (NCI) Surveillance, Epidemiology, and End Results-18 (SEER-18) Program collects cancer incidence data from population-based cancer registries. The file includes information from 18 high-quality, population-based registries (SEER-18: Alaska, Connecticut, Detroit, Atlanta, Greater Georgia, Rural Georgia, San Francisco-Oakland and San Jose-Monterey California, Greater California, Hawaii, Iowa, Kentucky, Los Angeles California, Louisiana, New Mexico, New Jersey, Washington [Seattle and Puget Sound region] and Utah) covering approximately 34.6% of the U.S. population (31.9% of Whites, 30.0% of African Americans, 44.0% Hispanics, 49.3% of American Indians and Alaska Natives, 57.5% of Asians, and 68.5% of Hawaiian/Pacific Islanders). Thyroid cancer cases diagnosed during 2011–2015 were ascertained from the SEER cancer incidence file which provides demographic and cancer diagnosis information for each case.

Data is abstracted from medical records at healthcare facilities, including hospitals, physician’s offices, and pathology laboratories, following the North American Association of Central Cancer Registries (NAACCR) data standards [1]. Since 1997, the PRCCR is funded by the CDC’s National Program of Cancer Registries (NPCR). Data in this analysis includes only microscopically confirmed cases with known age and sex. The PRCCR is Gold Certified by the NAACCR and recognized as a Registry for Surveillance by the NPCR. The PRCCR uses the coding standards of the SEER program and NAACCR. The criteria specified in the third revision of the International Classification of Diseases for Oncology (ICD-O-3) were used to select cases of invasive TC for the period 2011 to 2015.

For the purpose of the study, we categorized invasive TCs cases in two histologic subgroups: Papillary Thyroid Carcinoma (PTC; ICD-O-3 codes 8050, 8260, 8340–8341, 8343–8344, 8350) and “other” TC histologic types (Other TC; including all other ICD-O-3 TC codes). Crude and age-adjusted incidence rates were calculated using SEER*Stat software v.8.3.5. [35]. Data was analyzed by sex, age groups, racial/ethnic group, and histologic type. The incidence rates of invasive TC for the period 2011–2015 in PR and the US were compared. Denominators for PR’s incidence rates were sex-specific population estimates for PR based on the 2016 Vintage Population from US Census Bureau [36]. Annual incidence rates per 100,000 population were age-adjusted by the direct method to the 2000 US standard population using 19 age-categories. We assessed racial/ethnic differences by sex using the Standardized Rate Ratio (SRR) and its 95% CI. SRRs were calculated using Stata v.15 software [37]. When fewer than 6 cases were reported, the numbers and rates are not presented because of the potential for statistically unreliable estimates and to protect confidentiality [1]. The Institutional Review Board of the University of Puerto Rico-Medical Sciences Campus approved the study.

## Results

During the period 2011–2015, there were 5175 incident cases of thyroid cancer reported to the PRCCR. Eighty-one percent (*n* = 4208) of cases were women, and only 19% (*n* = 967) were men; 45% of the cases were diagnosed among the population aged 40–59; and 94% of the cases (*n* = 4877) were papillary thyroid carcinomas (PTC). By comparison, during the same period, there were 65,528 cases of TC diagnosed in the US, 75% (*n* = 49,373) of which were diagnosed in women, 44% were diagnosed among men and women aged 40–59 years, and 90% (*n* = 58,665) were classified as PTC. Given the small proportion of cases classified as “other” TC histologic types, our analysis focus only on PTC.

**Fig. 1:**
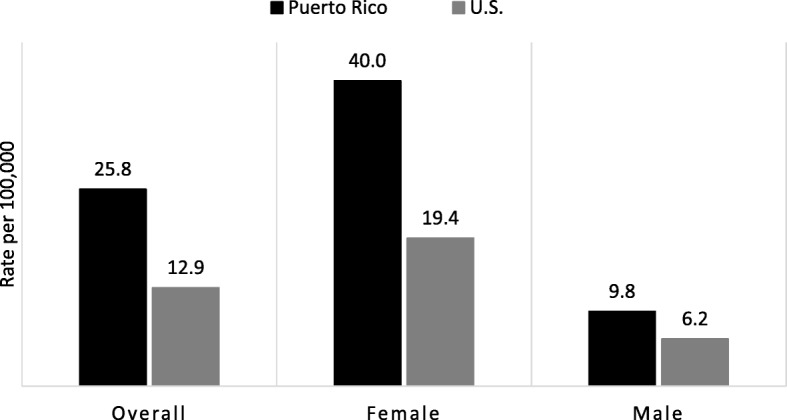
Age-Adjusted Incidence Rates of **PTC** by Sex in PR and US, 2011–2015

**Fig. 2:**
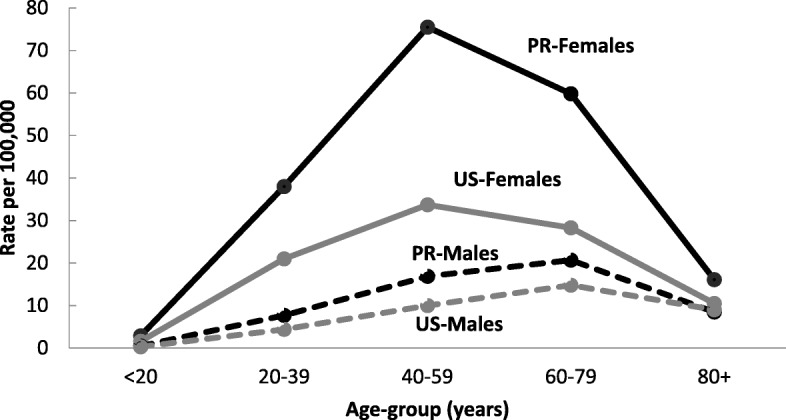
Age-Adjusted Incidence Rates of **PTC** by Age-Groups and Sex in PR and US, 2011–2015

Figure 1 shows the PR and US age-adjusted (2000 US Standard Population) incidence rates of PTC stratified by sex. The overall age-adjusted incidence rate of PTC was almost two-fold higher in PR than in the US (25.8/100,000 vs. 12.9/100,000). Among PR women, the incidence rate of PTC was 40.0/100,000 compared to 19.4/100,000 in US; whereas in men it was 9.8 and 6.2/100,000, respectively. Among PR and US women, age-specific incidence rates of PTC increased rapidly with age, peaked at age 40–59 years (PR: 75.5 vs. US: 33.7/100,000 women) declining sharply thereafter (Fig. 2). Among PR and US men, PTC age-specific rates peaked at an age a decade elder than in women, 60–79 years of age (PR: 20.7 vs. US: 14.8 /100,000).

**Fig. 3:**
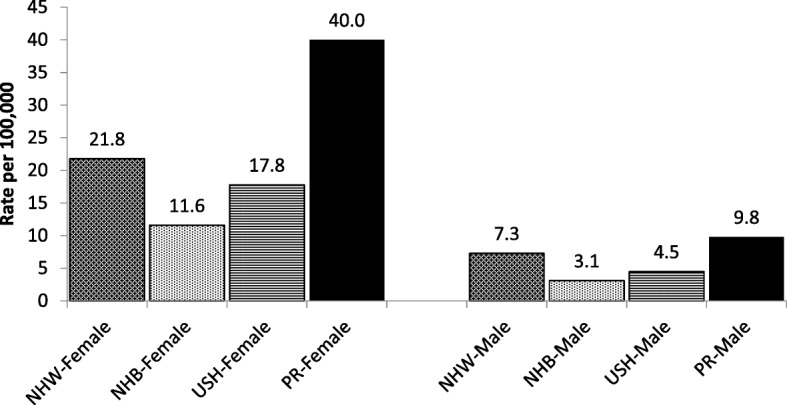
Age-Adjusted Incidence Rates of **PTC** by Race/Ethnicity, 2011–2015

Incidence rates of PTC in women and men living in PR were higher than those reported in other US racial/ethnic groups. Among women, PR had the highest age-adjusted incidence rate of PTC (40.0/100,000), followed by NHW (21.8/100,000), USH (17.8/100,000), and NHB (11.6/100,000) (Fig. 3). Similarly among men, PR had highest incidence rate of PTC (9.8/100,000) followed by NHW (7.3/100,000), USH (4.5/100,000), and NHB (3.1/100,000) (Fig. 3).

**Fig. 4:**
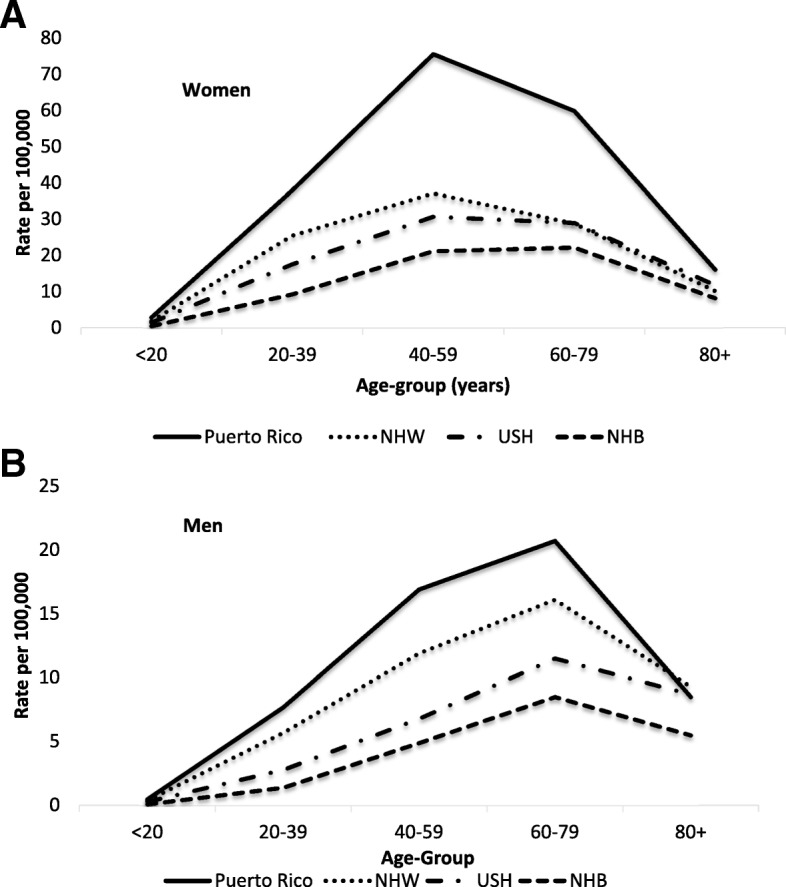
Age-Adjusted Incidence Rates of **PTC** by Sex and Race/Ethnicity in PR and US, 2011–2015. Women (**a**), Men (**b**)

**Fig. 5:**
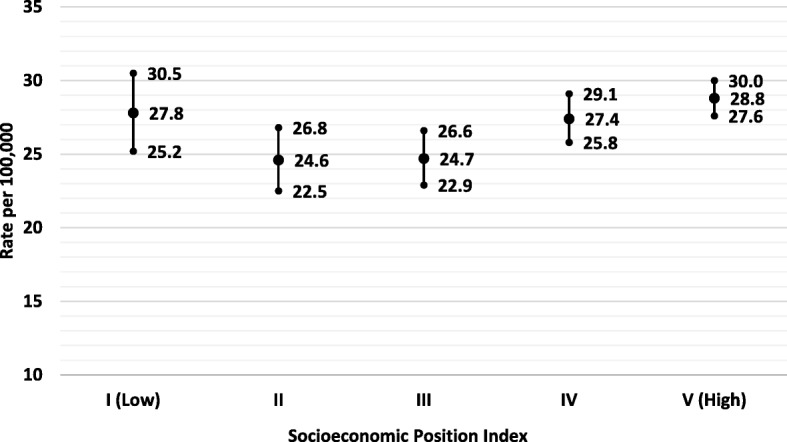
Age-Adjusted Incidence Rates (and 95% CI) for **TC** by Socioeconomic Position Index (SEP) in Puerto Rico, 2011–2015

Age-adjusted incidence rates of PTC by sex, age-group, and race/ethnicity are presented in Figs. 4 and 5. Compared to women of other racial/ethnic groups, PR women had the highest incidence rates; whereas, NHB women had the lowest incidence rates of PTC. This racial/ethnic differences were observed at all age groups. Among PR, NHW, and USH women, rates increased rapidly with aged, peaked at age 40–59 years (75.5, 37.1, and 30.7/100,000, respectively) declining rapidly thereafter. While among NHB women, rates increased steadily with age, peaked at age 60–79 years (22.2/100,000) declining thereafter (Fig. 4a). Similar to PR women, PR men had the highest incidence rates of PTC; whereas, NHB men had the lowest rate. These racial/ethnic differences were observed across all age groups. In men, all racial/ethnic groups showed similar pattern with age incidence rates of PTC increased with age, peaked at age 60–79 years and declined in older age groups (Fig. 4b).

Table 1 shows the standardized rate ratio (SRR) and its 95% confidence intervals assessing the difference in the risk of being diagnosed with PTC between PR and US race/ethic groups by sex and age group. PR women had 83% increased risk of being diagnosed with PCT than NHW women (SRR = 1.83; 95% CI 1.77–1.90), a 2.25-fold increased risk than USH (SRR = 2.25; 95% CI 2.17–2.34), and 3.45-fold increased risk than NHB women (SRR = 3.45; 95% CI 3.29–3.62) (Table 1). Similar difference were observed for men, PR men had a 34% increased risk of being diagnosed with PTC than NHW men (SRR = 1.34; 95% CI 1.25–1.44), 2.2-fold increased risk than USH (SRR = 2.19; 95% CI 2.01–2.38), and 3.2-fold higher risk than in NHB (SRR = 3.22, 95% CI (2.90–3.57) (Table 1).

**Table 1:** SRR of PTC by sex, age-group, and racial/ethnic groups during 2011–2015

	Age-adjusted rate				SRR (95% CI)		
	PR	NHW	USH	NHB	*PR* vs *NHW*^*a*^	*PR* vs *USH*^*a*^	*PR* vs *NHB*^*a*^
Women	40.0	21.8	17.8	11.6	1.83 (1.77–1.90)	2.25 (2.17–2.34)	3.45 (3.29–3.62)
< 20	2.9	1.9	1.5	0.5	1.51 (1.16–1.92)	1.92 (1.46–2.47)	5.79 (3.97–8.63)
20–39	38.0	25.5	17.6	9.3	1.49 (1.39–1.60)	2.17 (2.00–2.34)	4.08 (3.69–4.52)
40–59	75.5	37.1	30.7	21.2	2.03 (1.93–2.13)	2.45 (2.32–2.60)	3.56 (3.33–3.81)
60–79	59.8	28.8	29.0	22.2	2.08 (1.95–2.21)	2.06 (1.90–2.24)	2.69 (2.46–2.95)
80+	16.1	10.2	11.8	8.2	1.58 (1.21–2.00)	1.37 (1.00–1.85)	1.95 (1.38–2.78)
Men	9.8	7.3	4.5	3.1	1.34 (1.25–1.44)	2.19 (2.01–2.38)	3.22 (2.90–3.57)
< 20	0.5	0.4	0.3	0.1	1.45 (0.75–2.39)	1.72 (0.88–2.94)	5.17 (2.25–13.31)
20–39	7.7	5.7	2.8	1.4	1.35 (1.15–1.58)	2.71 (2.25–3.22)	5.37 (4.17–6.98)
40–59	16.9	11.9	6.8	4.9	1.42 (1.27–1.58)	2.50 (2.20–2.83)	3.43 (2.95–3.99)
60–79	20.7	16.1	11.5	8.5	1.29 (1.14–1.44)	1.80 (1.55–2.09)	2.42 (2.04–2.89)
80+	8.5	9.3	8.7	5.5	0.91 (0.58–1.32)	0.98 (0.58–1.57)	1.55 (0.86–2.84)

## Discussion

To our knowledge, this is the first study comparing the incidence rates of PTC between the population of PR and US racial/ethnic groups (NHW, NHB, and USH). In PR, consistent with studies conducted worldwide, the majority of TC cases were categorized as PTC (94% of cases). Our study showed significant differences in the incidence of PTC between the PR population and US racial/ethnic groups. Age-adjusted incidence rates of PTC were higher in PR residents, followed by NHW, USH, and NHB; higher in women than in men; and higher among those aged 40–59 years. Differences between PR residents and US racial/ethnic groups (NHW, USH, and NHB), were statistically significant (*p* < 0.05). These differences were observed in both women and men, and persisted across all age groups. Although results on “other” TC histologic types were not presented, our data showed no differences in the overall incidence of all “other” TC histologic types between PR and U.S. (1.5/100,000 vs. 1.5/100,000), respectively. Similarly, no sex or age differences in the incidence of “other” TC histologic types were observed between PR and U.S.

Reasons for the observed differences in PTC incidence rates between PR residents and US racial/ethnic groups are unknown and they are likely to be multifactorial. PR shares a high burden of some of the lifestyle and environmental factors than have been associated with TC. Based on data from the 2016 Behavioral Risk Factors Surveillance System and the 2015 US Diabetes Surveillance System, PR residents have higher prevalence rates of diabetes (PR 14.7% vs. US 9.1%); overweight and obesity (PR 66.5% vs. US 65.2%); lower rates of physical activity (150 min/week: PR 34.1% vs. US 51.3%); low intake of fruits (< 1 per day: PR 55.6% vs. US 39.7%) and low intake of vegetables (< 1 per day PR 24.5% vs. US 22.1%) [38]. In addition, a study conducted among adults aged 21–79 years residents of the San Juan metropolitan area showed high prevalence of metabolic syndrome (43.3%) among the study population [39]. The prevalence rate of metabolic syndrome was only slightly higher in men (45.3%) than in women (42.2%) and slightly higher in PR residents than among the US adult population [39]. However, the relationship between these factors and PTC risk is unclear. Nevertheless, like in other populations, it is unlikely that these factors might explain the rapid increase observed over the last three decades in the incidence of PTC in PR and worldwide. Neither, these factors explain the sex and age differences observed in PR and around the world.

Similar to other populations, the high PTC incidence rates observed in PR women and men, might be attributed to better access to medical care, the introduction of new diagnostic technologies, and the increase in medical surveillance, which results in overdiagnosis and overtreatment of subclinical indolent lesions [15, 18, 22, 40]. For the period 2003–2007, Vaccarella et al. estimated that overdiagnosis accounted for 90% of cases in South Korea of the cases diagnosed in women; 70 to 80% of cases in the United States, Italy, France and Australia; and 50% of cases in Japan, the Nordic countries, England and Scotland; whereas, in men overdiagnosis accounted for 70% of cases in France, Italy and South Korea; 45% in the United States and Australia; and less than 25% in the other countries analyzed [16]. Overdiagnosis is the result of multiple factors including patient, provider, healthcare system, and environment. These factors include patient’s socio-demographic characteristics; healthcare access; quality of cancer registration; mass-media influence; economic and medicolegal incentives; and medical education, among other; however most research has focus on the individual factors [41]. If overdiagnosis were the major driver of the observed differences in incidence of PTC between the PR population and the US racial/ethnic group population, we would expect to observe higher prevalence of those factors leading to overdiagnosis in PR than in the US.

The contribution of health insurance coverage and of access to healthcare to overdiagnosis and overtreatment is well documented [42]. Massachusetts has one of the highest health insurance coverage in the US. The government of PR, similar to the State of Massachusetts, implemented a Government Health Plan (GHP) in 1994 as a government-run program which provides healthcare services to the indigent and impoverished citizens of PR living below or at 250% federal poverty level. In 2016, 94% of the PR population and 97% of the Massachusetts’ population had health insurance coverage [42, 43]. In PR, approximately, 49% of the insured population receives healthcare coverage through Medicaid (Medicaid/CHIP and GHP) [32]. Massachusetts, the state with the highest health insurance coverage in the US, had the highest overall rate of TC (20.4/100,000) and male TC rate (10.7/100,000) and the second highest female rate of TC (30.2/100,000) in the US [44]. The expansion of Medicaid has been associated with increased access to healthcare and utilization of services. Massachusetts experienced an increase in the utilization of services and surgical procedures [42, 45–48], including a 26% increased rate of patients undergoing a thyroidectomy, 22% increased rate of neck dissection, 15% increased rate in admissions for pancreatic cancer and 67% increased rate of surgical resections for pancreatic cancer [42, 47]. The increase overtime in the utilization of these procedures was observed in both White and Non-White patients but the increase was faster in Non-White patients supporting the role of health insurance status in overdiagnosis and overtreatment and in reducing racial/ethnic and socioeconomic differences in access to care [42].

Other potential factors supporting the role of overdiagnosis to explain the differences in incidence of PTC between the PR population and US racial/ethnic groups, include: 1) the higher percentage of PTC lesions diagnosed in a localized stage in PR (76.3%) compared to the US (69.8%); 2) the large percentage of small lesions reported to the PRCCR between 2008 and 2015 (53% of lesions ≤1 cm and 33% of lesions 1.1 to 4 cm); and the large proportion of cases diagnosed using ultrasound guided procedures (> 47%) (SEER 18 Regs, Puerto Rico Central Cancer Registry 2018).

Higher socioeconomic status (SES) has been found to be associated with the risk of TC suggesting that SES might be a proxy measure of insurance status, access to healthcare, educational attainment, and other healthy behaviors [19, 49]. However, using the socioeconomic position (SEP) index developed by the PRCCR [50], we observed a “U” shape relationship between SES and the incidence of PTC in PR, with higher incidence rates observed in the lowest (I) and highest levels (IV and V) of the SEP index (Fig. 5). However, these differences were not statistically significant (*p* > 0.05). The lack of relationship between SES and the incidence of PTC among PR residents might be explained by the almost universal health insurance coverage in PR, which conducts to an increased access to healthcare which increases the opportunity for a diagnosis of PTC and results in overdiagnosis and overtreatment.

Furthermore, among cancer survivors in PR, TC was the fourth most common cancer type accounting 7.21% (4723 cases). Approximately, 83% (3905) of thyroid cancer survivors were women [51]. Among women, thyroid cancer accounted for 12.3% of all female cancer survivors [51]. Similarly in the US, TC survivors accounted for 14.6% of all cancer survivors. Women accounted for 78.3% (*n* = 630,660) of female cancer survivors and accounted for 7.7% of all female cancer survivors [52]. Finally, the cost of TC care in the US is estimated to reach $18–21 billion dollars in 2019 [53].

Recent data suggests that the rising incidence and overdiagnosis of TC may be starting to slow down. Since about 2015, there have been a variety of efforts to lessen the role of overdiagnosis in the increasing trend of TC. These efforts ranged from public education to guideline and diagnostic changes [54]. In 2015, the American Thyroid Association (ATA) recommended a risk-stratification approach to the use of fine needle aspiration biopsies of thyroid nodules. This guidelines recommend against biopsy of nodules less than 1 cm in size and for the first time the ATA guidelines recognized active surveillance as an alternative management strategy for patients with low-risk thyroid cancer [54]. Nevertheless, it is recognized that changing physician clinical behaviors is likely to be difficult and slow; therefore these efforts will require the development and implementation of evidence-based multilevel interventions in order to continue this reversed trend in faster manner [55]. In addition, in 2017 the United States Preventive Services Taskforce (USPSTF) recommended against TC screening in asymptomatic individuals, a grade ‘D’ recommendation [54]. Two recent studies concluded that from a value perspective, active surveillance was superior to surgery; whereas, a third study found that cost-effectiveness depends on variability in a patient’s perspective on quality of life [54].

## Conclusion

This is the first study comparing the incidence rates of PTC between the population of PR and US racial/ethnic groups (NHW, NHB, and USH). In PR, consistent with other studies conducted, the majority of TC cases were categorized as PTC (94% of cases). Our findings showed significant differences in the incidence of PTC between the PR population and US racial/ethnic groups. Similar to other studies, overdiagnosis seems to explain the high incidences rates. Further research is needed to address potential risk factors for PTC/TC that might explain this disparity in the island including: assessment of the extent of overdiagnosis in PR, the role of health insurance status and insurance type (GHP, Private Insurance, and uninsured), characteristics of the healthcare delivery system, and environmental and individual factors.

## Data Availability

The datasets generated and analyzed during the current study are not publicly available due to the confidentiality policy of the Puerto Rico Central Cancer Registry but are available from the corresponding author on reasonable request.

## Abbreviations

ATA: American Thyroid Association
GHP: Government Health Plan
ICD-O-3: International Classification of Diseases for Oncology 3rd Edition
NAACCR: North American Association of Central Cancer Registries
NHB: Non-Hispanic Blacks
NHW: Non-Hispanic Whites
NPCR: National Program of Cancer Registries
PR: Puerto Rico
PRCCR: Puerto Rico Central Cancer Registry
PTC: Papillary thyroid cancer
SEER: Surveillance Epidemiology and End Results Program
SES: Socioeconomic status
SRR: Standardized rate ratio
TC: Thyroid cancer
US: United States
USH: US Hispanics
USPSTF: United States Preventive Services Taskforce

## References

[1] Ferlay JS, Bray HR, Forman F, Mathers D, C. Parkin D. (2018). GLOBOCAN 2018: estimated cancer incidence, mortality, prevalence and disability-adjusted life years (DALYs) worldwide in.

[2] Puerto Rico Central Cancer Registry. Incidence Case File (April 2, 2018). 2018.

[3] Pellegriti G, Frasca F, Regalbuto C, Squatrito S, Vigneri R. Worldwide increasing incidence of thyroid cancer: update on epidemiology and risk factors. J Cancer Epidemiol. 2013. 10.1155/2013/965212 PMC3664492 23737785

[4] Reitzel LR, Nguyen N, Li N, Xu L, Regan SD, Sturgis EM (2014). Trends in thyroid Cancer incidence in Texas from 1995 to 2008 by socioeconomic status and race/ethnicity. Thyroid [Internet].

[5] La Vecchia C, Malvezzi M, Bosetti C, Garavello W, Bertuccio P, Levi F (2015). Thyroid cancer mortality and incidence: A global overview. Int J Cancer.

[6] Horn-Ross, Pamela L. Lichtensztajn, Daphne Y. Clarke, Cristina A. Dosiou, Chrysoula. Oakley-Girvan, Ingrid. Reynolds, Peggy. Gomez, Scarlett L. Nelson DO. Continued rapid increase in thyroid cancer incidence in California: trends by patient, tumor, and neighborhood characteristics. Cancer Epidemiol Biomark Prev 2014;23(6):1067–1079. 10.1158/1055-9965.EPI-13-1089 PMC4071298 24842625

[7] Davies L, Welch HG. Current thyroid cancer trends in the United States. JAMA Otolaryngol Head Neck Surg [Internet]. 2014 Apr [cited 2015 Oct 3];140(4):317–322. Available from: http://www.ncbi.nlm.nih.gov/pubmed/24557566. 10.1001/jamaoto.2014.1 24557566

[8] Pandeya N, McLeod DS, Balasubramaniam K, Baade PD, Youl PH, Bain CJ (2016). Increasing thyroid cancer incidence in Queensland, Australia 1982-2008 - true increase or overdiagnosis. Clin Endocrinol.

[9] Davies L, Welch HG. Increasing Incidence of Thyroid Cancer in the United States, 1973-2002. JAMA [Internet]. 2006 May 10 [cited 2015 Oct 20];295(18):2164. Available from: http://www.ncbi.nlm.nih.gov/pubmed/16684987. 10.1001/jama.295.18.2164 16684987

[10] Bray Freddie, Ferlay Jacques, Soerjomataram Isabelle, Siegel Rebecca L., Torre Lindsey A., Jemal Ahmedin (2018). Global cancer statistics 2018: GLOBOCAN estimates of incidence and mortality worldwide for 36 cancers in 185 countries. CA: A Cancer Journal for Clinicians.

[11] Hall SF, Irish J, Groome P, Griffiths R. Access, excess, and overdiagnosis: the case for thyroid cancer. Cancer Med [Internet]. 2014;3(1):154–161. Available from: http://www.pubmedcentral.nih.gov/articlerender.fcgi?artid=3930400&tool=pmcentrez&rendertype=abstract. [cited 2015 Oct 20]. 10.1002/cam4.184 PMC3930400 24408145

[12] Li Nan, Du Xianglin L., Reitzel Lorraine R., Xu Li, Sturgis Erich M. (2013). Impact of Enhanced Detection on the Increase in Thyroid Cancer Incidence in the United States: Review of Incidence Trends by Socioeconomic Status Within the Surveillance, Epidemiology, and End Results Registry, 1980–2008. Thyroid.

[13] Sosa JA, Hanna JW, Robinson KA, Lanman RB. Increases in thyroid nodule fine-needle aspirations, operations, and diagnoses of thyroid cancer in the United States. Surgery [Internet]. 2013 [cited 2015 Oct 20];154(6):1420–1426; discussion 1426–7. Available from: http://www.ncbi.nlm.nih.gov/pubmed/24094448. 10.1016/j.surg.2013.07.006 24094448

[14] Hoang JK, Choudhury KR, Eastwood JD, Esclamado RM, Lyman GH, Shattuck TM (2014). An exponential growth in incidence of thyroid cancer: trends and impact of CT imaging. Am J Neuroradiol.

[15] Franceschi S, Vaccarella S (2015). Thyroid cancer: an epidemic of disease or an epidemic of diagnosis?. Int J Cancer.

[16] Vaccarella S, Franceschi S, Bray F, Plummer M, Dal Maso L (2016). Worldwide thyroid-Cancer epidemic? The increasing impact of Overdiagnosis. N Engl J Med.

[17] Esserman PLJ, Thompson PIM, Reid PB, Peter P, Ransohoff PDF, Welch PHG, et al. Prescription for Change. Lancet Oncol. 2015;15(6). 10.1016/S1470-2045(13)70598-9 PMC4322920 24807866

[18] Udelsman R, Zhang Y (2014). The epidemic of thyroid Cancer in the United States: the role of endocrinologists and ultrasounds. Thyroid [Internet]..

[19] Guay B, Johnson-Obaseki S, McDonald JT (2014). CC& CM. Guey et al-thyroid 2014. Thyroid..

[20] Morris LGT, Sikora AG, Tosteson TD, Davies L (2013). The increasing incidence of thyroid Cancer: the influence of access to care. Thyroid.

[21] Harari A, Li N, Yeh MW (2014). Racial and socioeconomic disparities in presentation and outcomes of well-differentiated thyroid cancer. J Clin Endocrinol Metab.

[22] Vaccarella S, Dal Maso L, Laversanne M, Bray F, Plummer M, Franceschi S (2015). The impact of diagnostic changes on the rise in thyroid Cancer incidence: A population-based study in selected high-resource countries. Thyroid [Internet]..

[23] Kitahara et al., 2011; Kitahara CM, Schneider AB, Brenner AV in: Thun MJ, Linet MS,Cerhan JR, Haiman CA SD. ancer Epidemiology and Prevention. 4th ed. New York: Oxford University Press; 2018. 839–860 p.

[24] Cash SW, Ma H, Horn-Ross PL, Reynolds P, Canchola AJ, Sullivan-Halley J, et al. Recreational physical activity and risk of papillary thyroid cancer among women in the California Teachers Study. Cancer Epidemiol [Internet]. 2013 Feb [cited 2015 Oct 20];37(1):46–53. Available from: http://www.pubmedcentral.nih.gov/articlerender.fcgi?artid=3543486&tool=pmcentrez&rendertype=abstract 10.1016/j.canep.2012.09.003 PMC3543486 23116823

[25] Siu S, McDonald JT, Rajaraman M, Franklin J, Paul T, Rachinsky I, Morrison D, Imran SA, Burrell S, Hart R, Driedger A, Badreddine M, Yoo J, Corsten M, van Uum S (2014). Is lower socioeconomic status associated with more advanced thyroid cancer stage at presentation? A study in two Canadian centers. Thyroid..

[26] Magreni A, Bann DV, Schubart JR, Goldenberg D (2015). The effects of race and ethnicity on thyroid cancer incidence. JAMA Otolaryngol - Head Neck Surg.

[27] Brito JP, Lew JI, Solorzano CC. Risk of thyroid cancer in Hispanics: a cohort study. Am Surg [Internet]. 2013 Feb [cited 2015 Oct 20];79(2):213–214. Available from: http://www.ncbi.nlm.nih.gov/pubmed/23336663. 23336663

[28] Weeks KS, Kahl AR, Lynch CF, Charlton ME (2018). Racial/ethnic differences in thyroid cancer incidence in the United States, 2007-2014. Cancer..

[29] Merrill RM, Harris JD, Merrill JG (2013). Differences in incidence rates and early detection of Cancer among non-Hispanics and Hispanics whites in the United States. Ethn Dis..

[30] Pinheiro P. S., Sherman R. L., Trapido E. J., Fleming L. E., Huang Y., Gomez-Marin O., Lee D. (2009). Cancer Incidence in First Generation U.S. Hispanics: Cubans, Mexicans, Puerto Ricans, and New Latinos. Cancer Epidemiology Biomarkers & Prevention.

[31] Finlayson A, Barnes I, Sayeed S, McIver B, Beral V, Ali R (2014). Incidence of thyroid cancer in England by ethnic group, 2001–2007. British Journal of Cancer.

[32] US Census Bureau. American Community Survey 1-Year Estimates. 2015.

[33] US Census Bureau. American Community Survey 5-Year Estimates. 2015.

[34] Surveillance, Epidemiology, and End Results (SEER) Program (www.seer.cancer.gov) SEER*Stat Database: Incidence - SEER 18 Regs Research Data + Hurricane Katrina Impacted Louisiana Cases, Nov 2017 Sub (2000–2015) − Linke based on the N 2017 submission. SEER 18 Registries. 2018.

[35] Surveillance Research Program NCIS software (www.seer.cancer.gov/seerstat). SEER*Stat software.

[36] United States Census. Vintage 2016 estimates series from the Population Division of the United States Census Bureau. 2016.

[37] StataCorp. Stata: Release 15. Statistical Software. College Station TSL. STATA; 2017.

[38] US Department of Health and Human Services C for DC and P. Behavioral Risk Factor Surveillance System Survey Data [Internet]. 2016. Available from: https://www.cdc.gov/diabetes/data/index.html.

[39] Pérez CM, Guzmán M, Ortiz AP, Estrella M, Valle Y, Pérez N, Haddock L, Suárez E. NIH Public Access. Ethn Dis 2008;18(4):434–41. PMC2717036 19157247

[40] Morris Luc G. T., Tuttle R. Michael, Davies Louise (2016). Changing Trends in the Incidence of Thyroid Cancer in the United States. JAMA Otolaryngology–Head & Neck Surgery.

[41] Sanabria A, Kowalski LP, Shah JP, Nixon IJ, Angelos P, Williams MD (2018). Growing incidence of thyroid carcinoma in recent years: factors underlying overdiagnosis. Head Neck.

[42] Loehrer AP, Murthy SS, Song Z, Lubitz CC, James BC (2017). Association of insurance expansion with surgical management of thyroid cancer. JAMA Surg..

[43] Henry J Kaiser Family Foundation. Disparities Policy-Puerto Rico: Fast Facts [Internet]. KFF. 2017. Available from: https://www.kff.org/disparities-policy/fact-sheet/puerto-rico-fast-facts/

[44] US Cancer Statistics Working Group. US Cancer Statistics Data Visualizations Tool, based on November 2017 submission data (1999-2015): US Department of Health and Human Services, Centers for Disease Control and Prevention and National Cancer Institute [Internet] 2018. Available from: www.cdc.gov/cancer/dataviz.

[45] Ellimoottil C, Miller S, Ayanian JZ, Miller DC (2014). Effect of insurance expansion on utilization of inpatient surgery. JAMA Surg.

[46] Loehrer AP, Song Z, Auchincloss HG, Hutter MM (2013). Massachusetts health care reform and reduced racial disparities in minimally invasive surgery. JAMA Surg..

[47] Loehrer Andrew P., Chang David C., Hutter Matthew M., Song Zirui, Lillemoe Keith D., Warshaw Andrew L., Ferrone Cristina R. (2015). Health Insurance Expansion and Treatment of Pancreatic Cancer: Does Increased Access Lead to Improved Care?. Journal of the American College of Surgeons.

[48] Loehrer AP, Hawkins AT, Auchincloss HG, Song Z, Hutter MM, Patel VI (2016). Impact of expanded insurance coverage on racial disparities in vascular disease: insights from Massachusetts. Ann Surg.

[49] Altekruse Sean, Das Anita, Cho Hyunsoon, Petkov Valentina, Yu Mandi (2015). Do US thyroid cancer incidence rates increase with socioeconomic status among people with health insurance? An observational study using SEER population-based data. BMJ Open.

[50] Torres-Cintrón M, Ortiz AP, Ortiz-Ortiz KJ, Figueroa-Vallés NR, Pérez-Irizarry J, Díaz-Medina G, et al. Using a socioeconomic position index to assess disparities in cancer incidence and mortality, Puerto Rico, 1995–2004. Prev Chronic Dis [Internet]. 2012;9:E15. Available from: http://www.pubmedcentral.nih.gov/articlerender.fcgi?artid=3298767&tool=pmcentrez&rendertype=abstract. [cited 2015 Oct 16]. PMC3298767 22172182

[51] Estrada-Merly N, Ramos-Cartagena JM, Agosto-Rosa H, Zayas-Martínez LM, Rodríguez-Reyes LE, Torres-Cintrón CR, Alvarado-Ortiz M, Zavala D, Tortolero-Luna G, Ortiz AP. Sobrevivientes de Cáncer en Puerto Rico: 1987–2014. Puerto Rico: San Juan; 2017.

[52] American Cancer Society. Cancer Treatment & Survivorship Facts & Figures 2016–2017 [Internet]. Atlanta; 2016. Available from: https://www.cancer.org/content/dam/cancer-org/research/cancer-facts-and-statistics/cancer-treatment-and-survivorship-facts-and-figures/cancer-treatment-and-survivorship-facts-and-figures-2016-2017.pdf.

[53] Aschebrook-Kilfoy B., Schechter R. B., Shih Y.-C. T., Kaplan E. L., Chiu B. C.- H., Angelos P., Grogan R. H. (2013). The Clinical and Economic Burden of a Sustained Increase in Thyroid Cancer Incidence. Cancer Epidemiology Biomarkers & Prevention.

[54] Roman BR, Morris LG, Davies L (2017). The thyroid cancer epidemic, 2017 perspective. Curr Opin Endocrinol Diabetes Obes.

[55] Reschovsky JD, Rich EC, Lake TK (2015). Factors contributing to variations in physicians’ use of evidence at the point of care: A conceptual model. J Gen Intern Med.

